# Tool for Rapid & Easy Identification of High Risk Diabetic Foot: Validation & Clinical Pilot of the Simplified 60 Second Diabetic Foot Screening Tool

**DOI:** 10.1371/journal.pone.0125578

**Published:** 2015-06-29

**Authors:** M. Gail Woodbury, R. Gary Sibbald, Brian Ostrow, Reneeka Persaud, Julia M. Lowe

**Affiliations:** 1 Queen’s University, Kingston, ON, Canada; 2 University of Toronto, Toronto, ON, Canada; 3 Suddie Hospital, Essequibo, Guyana; Sapienza, University of Rome, School of Medicine and Psycology, ITALY

## Abstract

**Background:**

Most diabetic foot amputations are caused by ulcers on the skin of the foot i.e. diabetic foot ulcers. Early identification of patients at high risk for diabetic foot ulcers is crucial. The ‘Simplified 60-Second Diabetic Foot Screening Tool’ has been designed to rapidly detect high risk diabetic feet, allowing for timely identification and referral of patients needing treatment. This study aimed to determine the clinical performance and inter-rater reliability of ‘Simplified 60 Second Diabetic Foot Screening Tool’ in order to evaluate its applicability for routine screening.

**Methods and Findings:**

The tool was independently tested by n=12 assessors with n=18 Guyanese patients with diabetes. Inter-rater reliability was assessed by calculating Cronbach’s alpha for each of the assessment items. A minimum value of 0.60 was considered acceptable. Reliability scores of the screening tool assessment items were: ‘monofilament test’ 0.98; ‘active ulcer’ 0.97; ‘previous amputation’ 0.97; ‘previous ulcer’ 0.97; ‘fixed ankle’ 0.91; ‘deformity’ 0.87; ‘callus’ 0.87; ‘absent pulses’ 0.87; ‘fixed toe’ 0.80; ‘blisters’ 0.77; ‘ingrown nail’ 0.72; and ‘fissures’ 0.55. The item ‘stiffness in the toe or ankle’ was removed as it was observed in only 1.3% of patients. The item ‘fissures’ was also removed due to low inter-rater reliability. Clinical performance was assessed via a pilot study utilizing the screening tool on n=1,266 patients in an acute care setting in Georgetown, Guyana. In total, 48% of patients either had existing diabetic foot ulcers or were found to be at high risk for developing ulcers.

**Conclusions:**

Clinicians in low and middle income countries such as Guyana can use the Simplified 60-Second Diabetic Screening Tool to facilitate early detection and appropriate treatment of diabetic foot ulcers. Implementation of this screening tool has the potential to decrease diabetes related disability and mortality.

## Introduction

The global rise in diabetes prevalence is associated with an increase in diabetes-related complications. One of the most common of these complications is diabetes related non-traumatic lower extremity amputations [1]. Most diabetic foot complications that lead to amputations arise from the formation of diabetic ulcers of the skin [2]. Early detection of diabetic foot ulcers (DFU) is therefore a crucial step in preventing lower-limb amputations in patients with diabetes.

In 2013, the International Diabetes Federation (IDF) estimated 382 million people were living with diabetes worldwide and projected this number to increase to 592 million by 2035 [3]. Historically, diabetes has been a disease linked with high income countries e.g. the United States, Canada. However, at present 80% of diabetes deaths globally occur in low and middle income countries (LMICs) [4]. These countries are constrained by limited resources, delayed and complex clinical presentations, lack of patient awareness, and often health care providers with little to no formal training on diabetic foot complications [5].

The prevalence of DFUs is increasing worldwide; particularly in rapidly developing countries in Asia, Africa, and South America [5]. Such ulcers are mostly neuropathic, and therefore preventable with simple education and preventive techniques [1], [5], [6], [7]. Up to a quarter of people with diabetes will develop a foot ulcer in their lifetime, and a lower limb is lost every 30 seconds worldwide as a result of diabetes [8], [3].

DFUs cause enormous emotional, physical damage with direct and indirect financial losses, as well as an increase in mortality to 43–50% [8], [9], [10]. Amputation occurs 10–30 times more often in people with diabetes than the general population, and mortality increases from 13–40% in the first year after amputation to 35–65% at 3 years and 39–80% at 5 years after amputation [8], [10], [11].

Guyana is a South American country with an estimated annual per capita income of $8,100 [12]. Guyana is tied with Belize for the highest diabetes prevalence in the North American and Caribbean region, with 15.9% of the adult population affected [3]. Diabetes complications are also 5^th^ leading cause of death in the country [13].

In 2008, a group of Canadians and Guyanese established the Guyana Diabetes and Foot Care Project [14]. The principal aim of the project was to reduce diabetic foot complications in Guyana, where high diabetic foot morbidity and mortality was present. At the time, DFUs were the most common cause of admission to the surgical ward at the national teaching hospital—Georgetown Public Hospital Corporation (GPHC). Before the project, 42% of patients with diabetic foot problems admitted to the GPHC surgical ward required amputation. Half of these amputations were major (above ankle) amputations.

Early identification of patients at high risk for DFUs was a top priority due to the clinical and economic burden of diabetic foot complications. Routine screening for patients with a high risk diabetic foot is a necessary step for preventive care referral and optimization of health care resource utilization. As 85% of lower limb diabetes-related amputations are preceded by a DFU, routine screening is imperative [15]. This is especially so in LMICs. Indeed, research by Nayaran et al. has shown that preventative foot care for high risk patients is one of the three most cost-savings and feasible diabetes interventions in LMICs; the others being glycemic control to achieve HbA1c < 9.0% and blood pressure control to achieve < 160/95 mmHg [16].

The purpose of this study was to refine and investigate the feasibility of the novel Simplified 60-Second Diabetic Foot Screening Tool for use as a simple test to identify people at high risk of foot ulcer in a LMIC setting. This was done through 1) determining the inter-rater reliability of the individual items in the tool when used by clinicians from a high as well as a LMIC and 2) pilot testing the tool at GPHC to identify practical implementation issues.

## Methods

As there is no IRB at GPHC, permission to perform this study was obtained from. Dr Madan Rambaran, Director of the Institute of Health Sciences Education Georgetown Public Hospital Corporation. Procedures were in accordance with the Helsinki Declaration of 1975, as amended in 2008. The study was considered low risk for patient harm as no intervention was involved, the examination was already a part of routine care but took place at a special research visit and no personally identifying details were recorded. As literacy was a concern (according to UNICEF adult literacy is about 85%) written consent was not obtained. Instead, verbal consent was obtained in local dialect by Guyanese members of the team. To avoid hypoglycaemia while waiting, subjects were provided with a meal, individually thanked and also received a certificate of appreciation

### Tool Development

Diabetic foot screen risk factors have been well established in the literature. The International Wound Group for the Diabetic Foot (IWGDF), an IDF sponsored collaborative, summarized previously published factors in its risk classification system. These include: peripheral neuropathy (without or with deformity), peripheral arterial occlusive disease, and previous ulcer/amputation(s) [17]. The IWGDF also recommends that patients be categorized into four risk levels after examination with corresponding recommended follow-up schedules.

The Foot Care Interest Group of the American Diabetes Association has also proposed a number of risk factor items to be included in comprehensive foot examinations. These items include: patient history, dermatological and musculoskeletal inspection, neurological examination assessed via monofilaments and tuning fork, and vascular assessment [18]. The Group concluded that individuals with no identified risk factors should be re-examined annually, while those with risk factors should have more frequent specialized examination depending on the identified criteria.

To help primary care clinicians identify the high risk diabetic foot, Inlow et al identified risk criteria that could be assessed in approximately 60-seconds [19]. This ‘Inlow 60-Second Screen’ was later incorporated into a bedside tool with 12 items, a scoring system, and an overall score ranging from 0 to 23 [20]. This tool has been validated in several healthcare settings [19], [20]. Validation studies have identified the Inlow 60-Second Screen to have both excellent inter-rater and intra-rater reliability in long term care and dialysis unit settings [21]. However, in complex continuing care settings it had yielded a somewhat lower inter-rater reliability coefficient of 0.61 [22].

The Inlow 60-Second Screen was field tested by the study team in the Guyanese setting, but was found scoring too complex to be completed in a timely manner. Carreau et al echoed our field observations, demonstrating that the Inlow 60-Second Screen in fact required 7 minutes on average to complete, with a range of 2–21 minutes [22]. As a result, one of the authors of the Inlow 60-Second Screen (RGS) developed a modified tool from the literature using components of the Inlow tool as a foundation [19]. This novel tool is named the‘Simplified 60-Second Diabetic Foot Screening Tool’ and was the tool used in this study (Table 1). A 10g Semmmes- Weinstein monofilament was used for monofilament testing.

**Table 1:** Simplified 60-Second Screen for the HIGH-RISK DIABETIC FOOT 2012.

**Name:__________________________________________**		**CHECK BOTH FEET**	
**ID#: ________ Phone #:_____________________**		(Circle correct response)	
**Facility: _______________________________**			
**DOB (dd/mm/yy):_______/_______/_______**			
**Gender: M □ F □ Years with diabetes:________**		**“YES” on either foot = HIGH RISK**	
**Ethnicity: Black □ Asian □ Caucasian □ Mixed □ Other**		**LEFT**	**RIGHT**
**Date of Exam (dd/mm/yy): ______/______/______**			
**HISTORY**	**1**. Previous ulcer	NO YES	NO YES
	**2**. Previous amputation	NO YES	NO YES
**PHYSICAL EXAM**	**3**. Deformity	NO YES	NO YES
	**4**. Ingrown toenail (thickened nail fold)	NO YES	NO YES
	**5**.Absent pedal pulses (Dorsalis Pedis and/ or Posterior Tibial)	NO YES	NO YES
**FOOT LESIONS** *Remember to check 4^th^ and 5^th^ web spaces/nails for fungal infection and check for inappropriate footwear*.	**6**. Active ulcer	NO YES	NO YES
	**7**. Blisters	NO YES	NO YES
	**8**. Calluses (thick scale on plantar skin)	NO YES	NO YES
	**9**. Fissure (linear crack)	NO YES	NO YES
**NEUROPATHY** *MORE THAN 4/10 SITES LACKING FEELING =* ***“YES”***	**10**. Monofilament exam **(record negative reaction):**	NO YES	NO YES
	**a)**Right______/10 negatives		
	(4 negatives = Yes)		
	**b)** Left_______/10 negatives	**Total # of YES:_____**	**Total # of YES: ____**
	(4 negatives = Yes)		
**PLAN**			
**a) POSITIVE SCREEN-** Results when there are one or more “Yes” responses. **Refer to a foot specialist or team for prevention, treatment and follow up.** (Bony deformity, current ulcer, absent pulse are most urgent). These individuals are at increased risk of a foot ulcer and/or infection. Patients should be educated on what changes to observe and report, while waiting for the specialist appointment.			
**Referral to:** ____________________________ **Appointment time:**_______________________			
**b) NEGATIVE SCREEN**- Results when there are all “No” responses. **No referral required.** Educate patient to report any new changes to their healthcare provider and re-examine in 1 year.			
**One Year Date for Re-Examination (dd/mm/yy):________/________/________**			
**Completed By: __________________________ Date: _________________________________**			
**Additional Note:**			
For **POSITIVE SCREEN**, in addition to referral plan above, **positive risk factors** can be linked to the care recommendations in “Root Risk Classification and Follow- Up Guide” table on the bottom of reverse side. Local referral patterns may vary depending on expertise and available resources			

The first version of the Simplified 60-Second Diabetic Foot Screen contained the following items: ‘previous amputation’, ‘deformity’, ‘palpation for pulses’, ‘active ulcer’, ‘ingrown toenail’, ‘callus’, ‘blisters’, ‘fissure’, and ‘monofilament testing for neuropathy’. The scoring system of the Inlow tool was replaced with a referral pathway for a comprehensive foot assessment if any tool item was positive. The timing of the follow-up referral is based on the IWGDF 2008 recommendations. Follow-up scheduling is determined by the number of positive items recorded during the assessment:

- 1–2 month—previous ulcer, previous amputation, active ulcer, ingrown toenail
- 3–4 months—deformity, peripheral vascular disease, absent pulse
- 6 months—neuropathy (4/10 negatives on monofilaments, callus, blister)
- 12 months—no positive findings.

### Validation

Screening tools used in clinical practice require validation. It was important to establish inter-rater reliability as it was intended that many types of clinicians would use the Simplified 60-Second Diabetic Foot Screening Tool. It was determined that eighteen test participants were required based on the following criteria [23]:

- Six or more assessors
- Minimum acceptable reliability value of 0.60
- α-level of 0.05,
- β-level of 0.20, and
- Expected reliability value for the population of 0.80

Eighteen Guyanese persons with diabetes from the GPHC diabetes foot clinic were selected as test participants. A Guyanese physician (IB) who was familiar with the patients of the clinic selected the participants to represent the full range of the items on the Simplified 60-Second Diabetic Foot Screening Tool i.e. from patients with minimal foot involvement to patients with neuropathy, foot ulcers, callus, blisters, fissures and partial amputations.

Twelve clinical assessors participated in the inter-rater reliability study: six Canadian clinicians (3 physicians, 1 nurse, 1 nurse/certified diabetes educator, 1 chiropodist) experienced in screening the diabetic foot, and six Guyanese clinicians (2 physicians, 2 nurses, 1 physical therapist, 1 dietitian) who were selected and trained as key opinion leaders (KOLs) for the Project. The six Guyanese KOLs had received an 8-month training course through the International Interprofessional Wound Care Course at the University of Toronto. In addition, they received skills training and interprofessional care preceptorships by the Canadian team in both Canadian and Guyanese clinical settings to develop expertise in the management of the diabetic foot. All assessors performed each screen independently; that is without knowledge of the results of each other, and by having each of the 12 assessors assessing every participant independently.

Inter-rater reliability was calculated for all 18 test participants (in total 216 examinations) with Canadian and Guyanese raters grouped together, and then a separate analysis for the Canadian (108 examinations) and Guyanese (108 examinations) assessors [S1 Data]. Cronbach’s alpha was utilized as a measure of consistency among the assessors (rather than among items in the screening tool) [24]. The value deemed high enough for an acceptable result was 0.60, as it was considered the lowest acceptable value for the sample size determination.

### Pilot Testing

For the clinical pilot test, the Simplified 60-Second Diabetic Foot Screen was used by Guyanese clinicians to test 1,222 persons with diabetes in the GPHC medical diabetes outpatient clinic between 2008 and 2010 [S2 Data]. Any patient with a positive screening item was referred to the diabetes foot clinic at the GPHC.

## Results

### Inter-rater Reliability

Based on Cronbach’s alpha, the majority of the items were reliable for the combined group of assessors, as well as for Canadians and the Guyanese assessors separately. However, the item ‘*fissures*’ was borderline at 0.55 and has been removed from the tool

### Pilot Testing

The pilot test identified problems with the layout of the tool on the page and the design was changed as a result.

Table 2 illustrates the pilot test results. In total 47.2% of screened individuals already had ulcers or were considered high risk and were referred to the diabetic foot centre. The percentage of the 1,163 persons who tested positively for each item is also shown (no data was entered for 59 people). Many persons had more than one positive item. 9% percent of screened individuals had an active foot ulcer that was not detected previously and was not being treated (Table 3).

**Table 2:** Inter-rater reliability of the individual items in the Simplified 60-Second Diabetes Foot Screen Tool for the total group of raters and for the Canadian and Guyanese raters separately.

Simplified 60-second screen items	Canadians & Guyanese	Canadians	Guyanese
Previous Ulcer	.966	.975	.942
Previous Amputation	.969	.920	.948
Deformity	.874	.833	.665
Absent Pulses	.868	.828	.669
Fixed Ankle	.909	.909	.759
Fixed Toe	.798	.600	.696
Active Ulcer	.971	.923	.961
Ingrown Nail	.723	.481	.636
Callus	.874	.882	.690
Blisters	.768	.704	.587
Fissures	.553	.245	.415
Monofilament Test R foot	.983	.966	.971
Monofilament Test L foot	.978	.966	.955

**Table 3:** Simplified 60-Second Diabetes Foot Screen Tool Clinical Pilot: Percentage of 1,163* persons with diabetes who tested positively for each of the items.

Item	Present %	Absent %
Previous Ulcer	13	87
Previous Amputation	4	96
Deformity	8	92
Absent Pulses	11	89
Stiffness Ankle	4	96
Stiffness Toe	3	9.7
Active Diabetic Foot Ulcer	9	91
Ingrown Toenail	20	80
Callus	20	80
Blisters	4	96
Fissures	13	87
Neuropathy (via Monofilament Test)	29	71
Referred to Diabetic Foot Centre	45	55

* No data entered on 59 people.

The items ‘stiffness of the ankle’ and ‘stiffness of the toe’ were each present in 4% or less of participants and were therefore eliminated from the tool [14].

The 340 nurses and other healthcare professionals who received training with this tool have successfully implemented its use in 89 community clinics and regional healthcare facilities throughout Guyana.

## Discussion

The Simplified 60-Second Diabetic Foot Screening Tool was developed for and validated in a high risk population in a low and middle income setting [25]. By comparison, the Inlow tool was designed for use by family physicians in Canada in private practice. The Simplified 60-Second Diabetic Foot Screening Tool has been refined to maximize time efficiency in routine clinical practice. By contrast, the Inlow tool gives each item an equal scoring weight and within an item has weighted differences corresponding to a summative score that does not lead to a specific clinical action. Each of these tools may have different utility. The two tools are compared in greater detail in Table 4.

**Table 4:** Differences between the Inlow 60-second screening tool and the Simplified 60-Second Diabetes Foot Screen Tool.

Items: Comments	Inlow 60-second tool	Simplified 60-second tool
Development: both versions are based on clinical experience and best practice guidelines	Risk criteria that could be assessed in approximately 60-seconds were identified by Dr. Shane Inlow [12]. Bedside tool and scoring were developed/revised to match guideline recommendations [11]	Developed based on risk criteria established in international clinical practice guidelines and pilot-tested in the present study [5]
Validation: both versions have some validation	1. Inter-rater and intra-rater reliability, preliminary predictive validity determined in LTC and Dialysis unit [12]2. Inter-rater reliability determined in Complex Continuing Care [13]	Inter-rater reliability (this paper)
Tool scoring: Scoring of Inlow version is more complicated and subject to mathematical errors	Each item is given a score and the total is determined for each foot, then linked to risk categories (based on the International Guidelines), to a rescreening schedule, and to clinical setting specific considerations	If any one item is positive, referral is made to Diabetic Foot Centre
Tool instructions: Both tools have specific instructions to aid their correct completion and interpretation	These are attached with the tool	These are attached with the tool
Time for completing: Inlow takes too long for many settings	Not tested in most settings. Average of 7 minutes by Advanced Practice Nurses in Complex Continuing Care [13]	Assumed to take about 60-seconds
Settings for use of the tool: Because of the nature of the tools, they could be used in different settings, depending upon needs.	Could be used by physicians, nurses to describe patient characteristics as well as to determine risk	Could be used by physicians, nurses to describe patient characteristics as well as to determine risk/ need for referral
Educational requirements: should be done for both tools	Yes	Yes

The study’s findings resulted in modifications to the tool, which led to development of the Simplified 60-Second Diabetic Foot Screen (see S1). The modifications included the addition of more specific anchors for a ‘yes’ response. The low value of Cronbach’s alpha for the ‘fissures’ item confirmed that it is difficult for someone without advanced knowledge. Clinically, it is difficult to accurately determine if the fissure contains the required dermal base and not a more superficial epidermal base, especially on the heel region. The heel epidermis is extremely thick and intra-epidermal superficial linear cracks can be deceiving. Most participants with true dermal based fissures have coexisting neuropathy. Because of these issues the item was eliminated.

The strengths of the reliability study include the methodological selection of test participants based on the need to represent each of the clinical criteria in the tool, the independence of the assessments, and the completion of all of the examinations. In addition, the assessors of the tool were representative of the inter-professional group that may perform this type of assessment in real life clinical practice: physicians, nurses and allied health professionals. Limitations of the study include the selection of participants from only one specific setting, i.e. a tertiary hospital LMIC setting in Georgetown, Guyana, and our inability to follow a cohort to confirm the ability of the test to identify people who will develop foot ulcers, due to few patients to date having had repeat examinations.

Diabetic foot disease in LMICs leads to significant disability, deprivation and mortality. There is a need for well-planned interventional programs to address DFUs in LMICs. The Simplified 60-Second Diabetic Foot Screening Tool is designed to meet the need for structured identification and management of the high risk diabetic foot, a basic requirement for such programs. It is rooted in international clinical practice guidelines and provides a pathway for rapid and simple identification of people who need referral to a diabetes foot centre for early treatment. In addition, it also identifies patients in need of more intense education and management to prevent DFUs. The high inter-rater reliability of the remaining components of the tool demonstrates it to be a reproducible clinical tool with potential widespread utility.

This tool is also a prime example of the concept of reverse innovation, whereby innovated solutions are brought from low and middle income settings (e.g. Guyana) to high income ones (e.g. Canada). Though pilot tested in Guyana, the tool is now being used in clinics throughout North America. The Simplified 60-Second Diabetic Foot Screening Tool is user friendly and time efficient, facilitating adoption in routine clinical practice in all settings, be they high, middle or low income.

## Conclusions

Recognition of DFUs is imperative in the prevention of further diabetes-related complications and lower extremity amputations. The Simplified 60-Second Diabetic Screening Tool is a validated and pilot tested tool that enables the early, quick and reliable identification of diabetic skin ulceration in everyday clinical practice in high, middle or low income settings. Widespread adoption of the tool may ultimately result in a decrease in the amputation rate in patients with diabetes and thereby reducing the immense global burden of diabetes-related foot complications.

## Supporting Information

S1 Data Reliability. (XLSX)

S2 Data Analysis of Final entered 60 sec screen. (XLS)
